# Mono-(2-ethylhexyl) phthalate reversibly disrupts the blood-testis barrier in pubertal rats

**DOI:** 10.1093/toxsci/kfad116

**Published:** 2023-11-06

**Authors:** Richa Tiwary, John H Richburg

**Affiliations:** Division of Pharmacology and Toxicology, Center for Molecular and Cellular Toxicology, College of Pharmacy, The University of Texas at Austin, Austin, TX, USA; Division of Pharmacology and Toxicology, Center for Molecular and Cellular Toxicology, College of Pharmacy, The University of Texas at Austin, Austin, TX, USA

**Keywords:** phthalates, blood-testis barrier, pubertal rats

## Abstract

The blood-testis barrier (BTB) is constituted by tight junctions between adjacent Sertoli cells (SCs) that create a specialized adluminal microenvironment to foster the development of spermatocytes and spermatids. The BTB is a well-studied target of numerous environmental toxicants, including di-(2-ethylhexyl) phthalate (DEHP), a compound widely used in various consumer products. Mono-(2-ethylhexyl) phthalate (MEHP) is the active toxic metabolite of DEHP that has long been recognized in postnatal rodents to disrupt SC function. This study evaluates the impact of MEHP on the integrity of the BTB in both pubertal and adult rats and the signal transduction pathways known to be involved in the disruption of the BTB. Treatment of prepubertal rats with 700 mg/kg MEHP for 24 h functionally disrupted the BTB integrity. A similar treatment of adult rats with MEHP did not disrupt the integrity of the BTB. The observed disruption of the BTB integrity in the MEHP-treated prepubertal rats occurred concomitantly with a decreased expression and mislocalization of both the ZO1 and occludin tight junction-associated proteins, as well as sloughing of spermatocytes and spermatids. At this same time, MEHP treatment induced a transient surge of p44/42 mitogen-activated protein kinase (MAPK) pathway. Interestingly, after a recovery period of 5 weeks, the BTB recovered and was functionally intact. This is the first report to indicate that acute MEHP exposure of prepubertal rats, but not adult rats, disrupts the functional integrity of the BTB and that this effect on the BTB is reversible.

Spermatogenesis is the process of germ cell proliferation and differentiation in the male testis that is closely regulated by Sertoli cells (SCs). It is well known that SCs play a crucial role in the creation of tight junctions between adjacent SCs, resulting in the formation of a specialized adluminal microenvironment required for the development of spermatocytes and spermatids. Transmembrane components of these tight junctions form an impermeable barrier between adjacent SCs, known as the blood-testis barrier (BTB), thereby creating distinct basal and adluminal compartments (Russell and Peterson, 1985).

Recently, we reported that acute mono-(2-ethylhexyl) phthalate (MEHP) exposure in pubertal rats leads to a significant infiltration of peritubular macrophages, which are known to play a role in the stimulation of the spermatogonium stem cell niche (Gillette *et al.*, 2021). We have hypothesized that the influx of the peritubular macrophages may play a role in the recovery of spermatogenesis after toxicant-induced testicular injury. However, the signaling mechanisms responsible for instigating the increased number of peritubular macrophages in the testis are not understood.

The completion of the BTB occurs early in animals during the postnatal period before the development of immunotolerance. As a result, the germ cells are not recognized as “self” by the immune system but instead would be recognized as foreign to the immune cells outside the BTB barrier (Mruk and Cheng, 2015). Therefore, a BTB disruption could allow germ cell antigens to “leak” into the basal compartment and be detected by immune cells that could express chemokines to stimulate the influx of innate immune cells, such as peritubular macrophages, into the testis. Therefore, understanding if MEHP can alter the functionality of the BTB is necessary for discerning the mechanism for the observed influx in peritubular macrophages.

Disruption of the functional integrity of the BTB can lead to premature germ cell loss and, ultimately, infertility (Russell and Peterson, 1985). Several major protein components of tight junctions in SCs have been identified: claudin 11, occludin, and zonula occludens (ZO-1). ZO-1 is a linker protein as it has binding sites for both claudin and occludin, linking the transmembrane tight junction proteins to SCs (Gilula and Fawcett, 1976). Thus, tight junctions play a critical role, and any disruption would lead to impaired spermatogenesis.

Phthalates, a class of synthetic chemicals used in various consumer products, have been shown to exert adverse effects on SCs, which play a pivotal role in the maintenance of testicular microenvironment and sperm development. They disrupt SC function, reduce essential factor production like anti-Müllerian hormone and inhibin B, which are crucial for sperm maturation; impair structural integrity, and interfere with the endocrine signaling pathways necessary for SC function, potentially disrupting the hormonal regulation of spermatogenesis (Corpuz-Hilsabeck and Culty, 2023). This disrupts male fertility, highlighting the need for more awareness and regulation of phthalate exposure.

MEHP is primarily a SC toxicant (Boekelheide *et al.*, 1989). MEHP-induced injury to SCs causes a functional disruption in their ability to maintain the specialized adluminal niche required for developing germ cells (Richburg *et al.*, 1999; Yao *et al.*, 2009). Although few reports have investigated that chronic (di-(2-ethylhexyl) phthalate [DEHP]) phthalate exposure leads to a reduction in expression levels of gap and tight junctions in mice, no study has explored acute MEHP-induced disruption on BTB in pubertal and adult rats (Shen *et al.*, 2017; Zhao *et al.*, 2023).

Previous studies from our lab have reported that acute MEHP exposure leads to a transient influx of macrophages in pubertal rats but not in adults (Murphy *et al.*, 2014; Voss *et al.*, 2018). Additionally, we have previously revealed that acute MEHP exposure instigates spermatocyte apoptosis in pubertal but not adult rats (Murphy *et al.*, 2014; Voss *et al.*, 2018). In this study, we evaluated if MEHP-induced apoptosis of spermatocytes is associated with perturbed BTB in pubertal rats and whether a disruption of the BTB by MEHP is reversible after a recovery period.

## Materials and methods

###  

####  

##### Animal care and treatments

Male Fischer CDF344 rats were purchased from Charles River. Animals were maintained in a controlled temperature (22°C ± 0.5°C) and lighting (12L:12D) environment and allowed to acclimate for 1 week before experimental procedures. Standard laboratory chow and water were supplied *ad libitum*. All animal procedures were performed according to the guidelines and approval of The University of Texas at Austin’s Institutional Animal Care and Use Committee, which abides by NIH Publications No. 8023, revised 1978.

In this study, comparisons between peripubertal and adult-aged animals were performed as apoptosis of spermatocytes was observed in peripubertal (PND 27) rats but not in adults (PND 65) (Murphy *et al.*, 2014). Exact age PND 27 or PND 65 male Fischer F344 rats (*n* = 7 for each treatment group) were treated with a single oral dose of MEHP (0.7 g/kg in corn oil, p.o.; 97.3% purity; Wako Chemicals) or an equivalent volume of vehicle (corn oil, 2 ml/kg, p.o.). While it’s acknowledged that the acute dose of MEHP (700 mg/kg) used in this study is not typically found in the environment, it’s important to note that the high-dose exposure model has a proven track record for rapid identification of endpoints due to its consistent and robust phenotype in the rodent. This specific dose aligns with extensive research conducted over many years (Gillette *et al.*, 2021; Murphy *et al.*, 2014; Voss *et al.*, 2018; Yao *et al.*, 2010), which laid the groundwork for our current study. Significantly, these previous studies have uncovered the fundamental mechanisms underlying the initiation of germ cell loss through death-receptor-triggered apoptosis.

At 24 h after the treatment, animals were euthanized using a ketamine (100 mg/ml) and xylazine (20 mg/ml) cocktail (0.1 mg/10 g body weight; Animal Health International). For some experiments, rats were allowed to recover from treatment for 5 weeks before euthanizing and assessing the recovery of BTB function. Animals were euthanized by CO_2_ asphyxiation followed by cervical dislocation.

##### Biotin tracer studies

The permeability of the BTB was assessed with a biotin tracer assay, as described previously (Meng *et al.*, 2005). Freshly prepared 10 mg/ml EZ-link Sulfo-NHS-LC-Biotin (Thermo Scientific, Catalog No. 21335) dissolved in PBS containing 1 mM CaCl_2_ was injected under tunica albuginea immediately after dissecting the testes. After 30 min of incubation at room temperature, testes were fixed in Bouin’s fixative and embedded in paraffin. Testis sections (10 µM) were prepared and incubated with streptavidin-Alexa Fluor 488 (1:250) for 30 min. All sections were imaged using a Nikon Eclipse microscope and captured with a Nikon Cool-SNAP digital camera. Images were processed and analyzed using NIS Elements software.

##### Immunofluorescence

Immunofluorescent analyses of tight junctions were performed on Bouin’s fixed paraffin-embedded testicular sections (5 µM). Heat treatment in a sodium citrate-based antigen retrieval system was used, followed by incubation in 3% hydrogen peroxide for 15 min to quench hydrogen peroxidase activity. Nonspecific binding was blocked using normal horse serum diluted 1:4 in PBS with 5% BSA (Bovine Sum Albumin). Testis sections were then incubated with anti-ZO-1 antibody (Cat # 617300; Invitrogen) diluted 1:100 blocking solution overnight at 4°C, followed by a 1-h room temperature incubation in Alexa Fluor 488-conjugated anti-rabbit antibody (1:500; Life Technologies). Anti-occludin antibody (Cat No. E6134R; Cell Signaling) diluted 1:200 in blocking solution overnight at 4°C, followed by incubation for 1 h at room temperature with Alexa Fluor 488-conjugated anti-mouse antibody (1:500; Life Technologies). Anti-caspase 3 antibody (Cat No. 9664L; Cell Signaling) diluted 1:100 in blocking solution overnight at 4°C, followed by a 1-h room temperature incubation in Alexa Fluor 488-conjugated anti-rabbit antibody (1:500; Life Technologies). Sections were mounted with VectaShield Mounting medium (Vector Labs). All sections were imaged as described above.

##### Histology

Standard periodic acid Schiff hematoxylin staining (PAS-H) protocols, as described previously, were followed (Voss *et al.*, 2018).

##### Western blot

Lysate from the tissue was prepared by homogenizing the frozen tissue section on ice in radioimmune precipitation assay (RIPA) buffer (PBS containing 1% Nonidet P-40, 0.5% [w/v] deoxycholic acid, 0.1% SDS, 1 mM PMSF, and 1 mM DTT) supplemented with complete mini protease inhibitor tablets (Roche Diagnostics). Protein samples were resolved on denaturing polyacrylamide gels, and transferred to nitrocellulose membrane (Whatman). Nonspecific binding was blocked with 5% milk (w/v) in 0.1% TBST. Membranes were then incubated with either phospho-JNK ½ (Cat No. 9255; Cell Signaling) or phospho-ERK (Cat No. 3192; Cell Signaling) or phospho-p38 (Cat No. 4511; Cell Signaling) or GAPDH antibodies (Cat No. 5174; Cell Signaling), followed by horseradish peroxidase-conjugated secondary antibodies (GE Healthcare). Peroxidase activity was detected by an enhanced chemiluminescence analysis system (GE Healthcare).

## Results

###  

#### MEHP disrupts the blood-testis barrier

**Figure 1.**
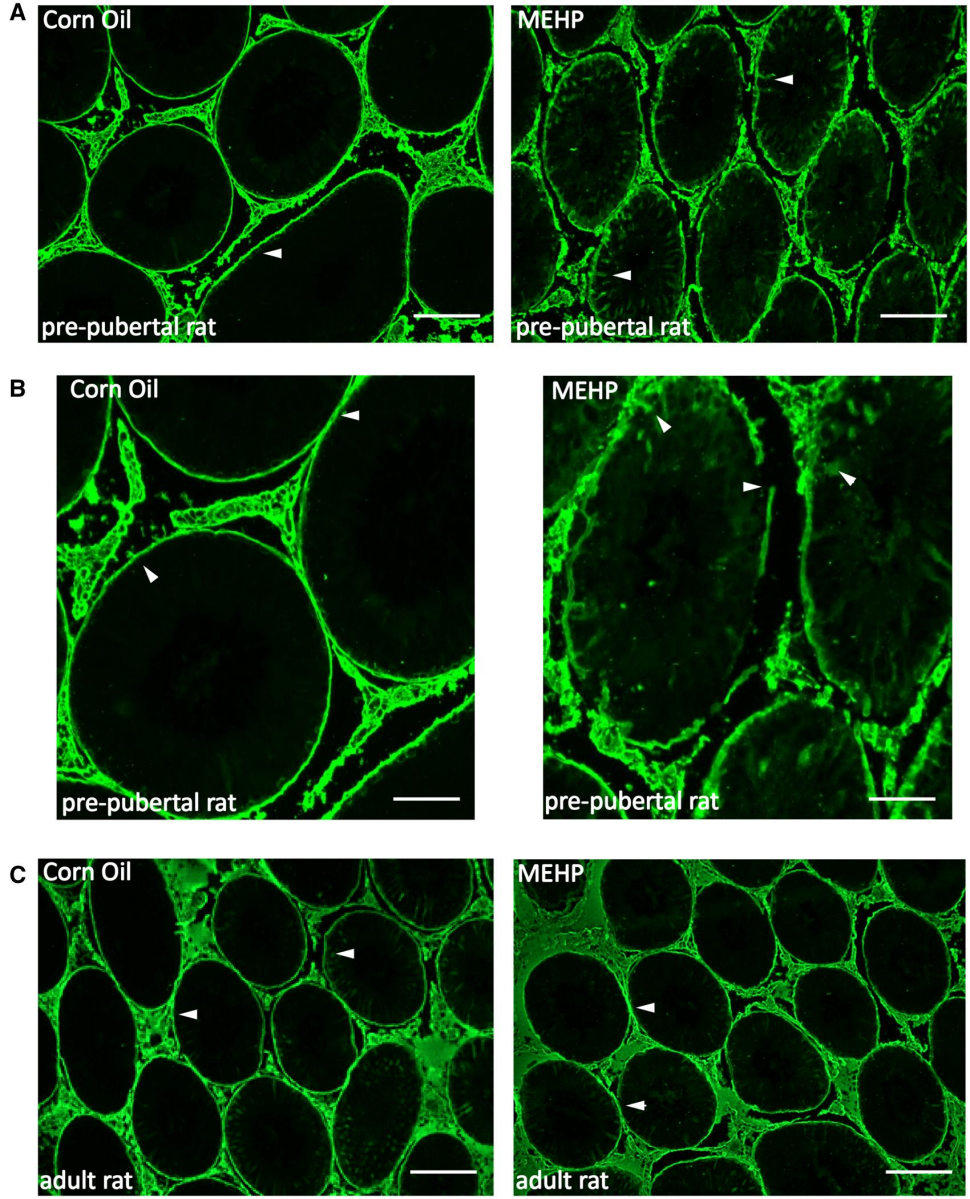
MEHP disrupts BTB. Biotin tracer assay to evaluate the integrity of BTB. Biotin is observed to permeate into the lumen of seminiferous tubules (indicated by arrowheads) only in MEHP (0.7g/kg) treated pre-pubertal rats (A) but not adults, where biotin is restricted to basal compartment (C). Higher magnification 20× image of biotin penetrating in the tubules of MEHP-treated group (B). Images are representative of *n* = 7 for each treatment. Scale bars = 50 μm.

The impact of MEHP on BTB integrity in pre-pubertal rats was assessed using the biotin tracer Sulfo-NHS-LC-Biotin penetration assay. Figure 1 shows that in the corn oil-treated groups in both peripubertal and adult rats (control, Figs. 1A and 1B), the BTB was intact as biotin was restricted to the interstitial area and basal compartment of the seminiferous epithelium whereas in the MEHP-treated groups, the biotin tracer was found to permeate into the adluminal space (arrowheads) of the seminiferous tubule in peripubertal rats (PND 27) (Fig. 1C) but not in adults (PND 65) (Fig. 1D).

#### Assessment of expression and localization of tight junction proteins after MEHP treatment in peripubertal rats

**Figure 2.**
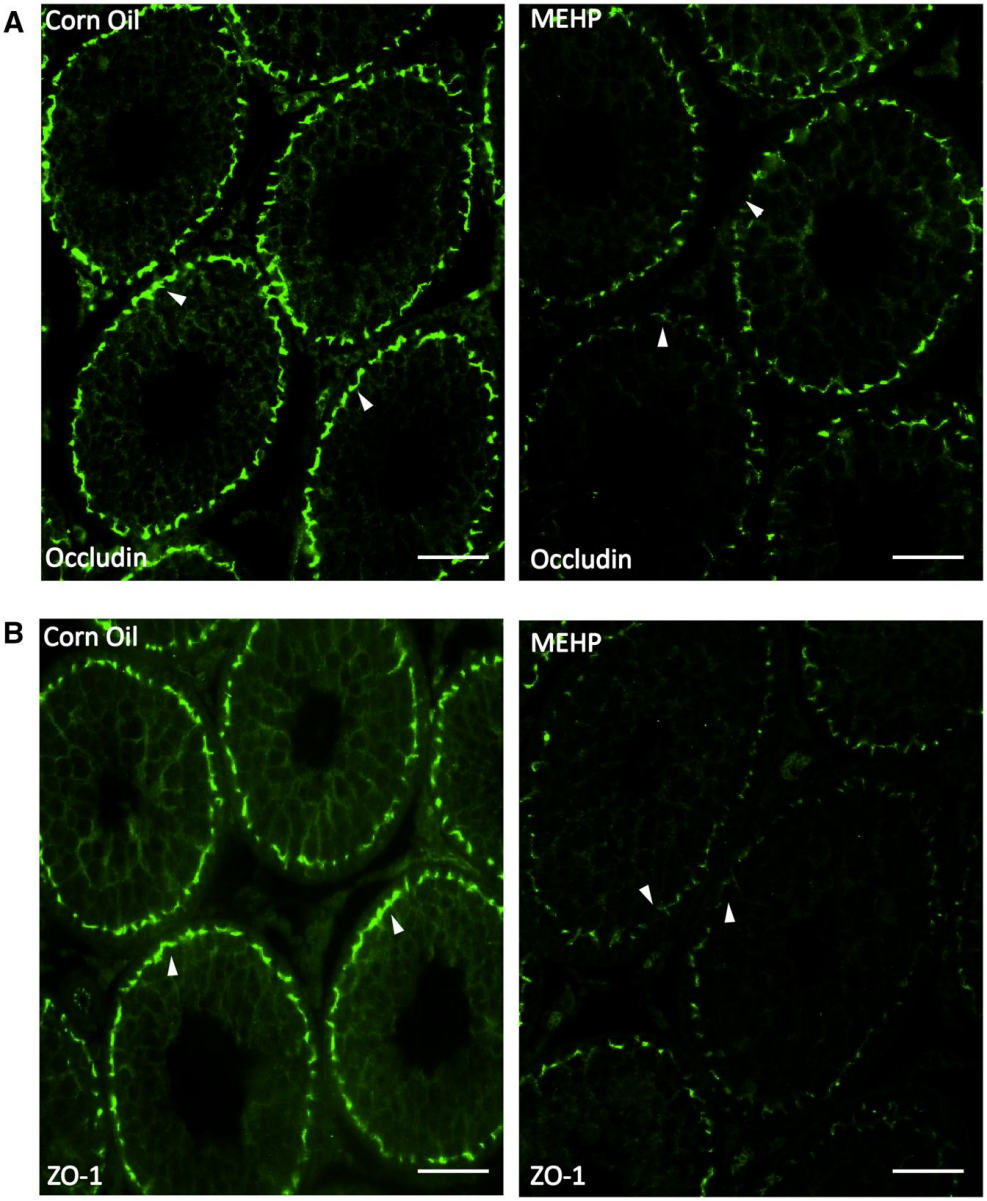
Expression and localization of tight junctions (Occludin, ZO-1) after MEHP treatment. Immunofluorescent staining of (A) Occludin and (B) ZO-1 depict linear and continuous patterns restricted to the basal region in testis sections (indicated by arrowheads) of the corn oil (control) group, whereas random and reduced expression levels are observed after 24 h of MEHP exposure in pubertal rats. Images are captured with 20× objective and represent *n* = 7 for each treatment. Scale bars = 50 μm.

The immunofluorescence data in Figure 2B shows that occludin in the corn oil (control)-treated groups of both PND27 and P65 rats (Figs. 2C and 2D, respectively) was restricted to the basal compartment in a linear continuous pattern (arrowheads) of seminiferous tubules at the basolateral membrane location of SCs. However, for the MEHP-treated groups, occludin was observed to be discontinuous and mis-localized, as well as with reduced expression levels. ZO1 was localized in a linear and continuous pattern in the basal compartment of seminiferous tubules in the corn oil (control)-treated group, whereas the localization was observed to be more irregular and with decreased expression levels in the MEHP-treated group (Fig. 2A).

#### MEHP-induced testicular damage and apoptosis of germ cells

**Figure 3.**
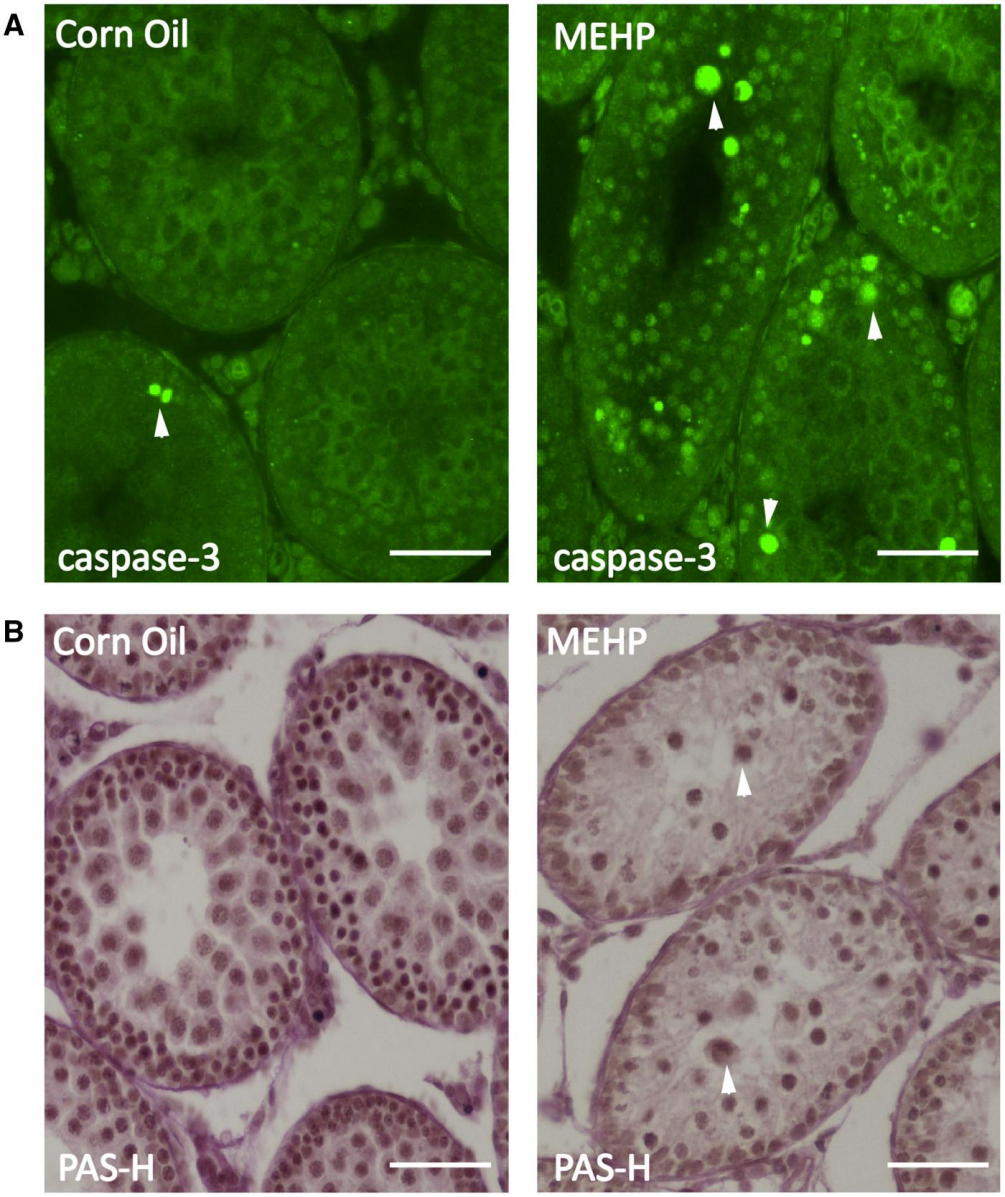
MEHP induces apoptosis of germ cells. Immunofluorescent staining of caspase 3 shows increased apoptosis of germ cells in MEHP-treated group in comparison to the corn oil group (A). Positively stained apoptotic cells are indicated by arrowheads. Periodic acid Schiff and hematoxylin stain (PAS-H) (3B) depicts normal spermatogenesis in all seminiferous tubules in testis sections of corn oil-treated (control) PND 28 Fischer rats. After 24 h of MEHP treatment, some seminiferous tubules depicted sloughing of the germ cells in the lumen (indicated by arrowheads) (B). Images are captured with 20× objective and representative of *n* = 7 for each treatment. Scale bars = 50 μm.

Caspase 3 immunofluorescent staining data show that exposure to MEHP leads to increased germ cell apoptosis in comparison to the control group (Fig. 3A). This finding is consistent with our previous reports (Murphy *et al.*, 2014; Voss *et al.*, 2018).

Evaluation of PAS-H-stained tissue cross-sections revealed normal spermatogenesis in all seminiferous tubules in the corn oil-treated (control) group (Fig. 3B). However, after MEHP treatment, some seminiferous tubules showed sloughing of the germ cells in the lumen (Fig. 3B).

#### MEHP regulates the MAPK pathway

**Figure 4.**
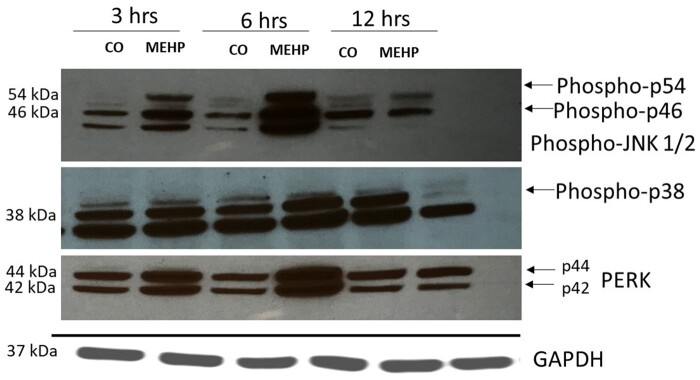
MEHP activates MAPK signaling pathway. Western blot analyses of whole testis of prepubertal rats treated orally with corn oil or MEHP (0.7g/kg) for 3, 6, and 12 h. MEHP-induced robust activation of MAPK signaling transduction activators-pJNK1/2, p-p38, pERK. GAPDH was used as a loading control. Images are representative of *n* = 7 for each treatment.

We further investigated if MEHP treatment in pre-pubertal rats induced changes in the activation levels of the MAPK signal transduction pathway. Western blot analyses show that MEHP treatment induced a robust increase in phosphorylated JNK1/2, phosphorylated p38, and phosphorylated ERK levels at 3 and 6 h after MEHP exposure, with peak activation at 6 h (Fig. 4). No changes were observed at 12 h after MEHP treatment.

#### MEHP induces reversible BTB disruption

**Figure 5.**
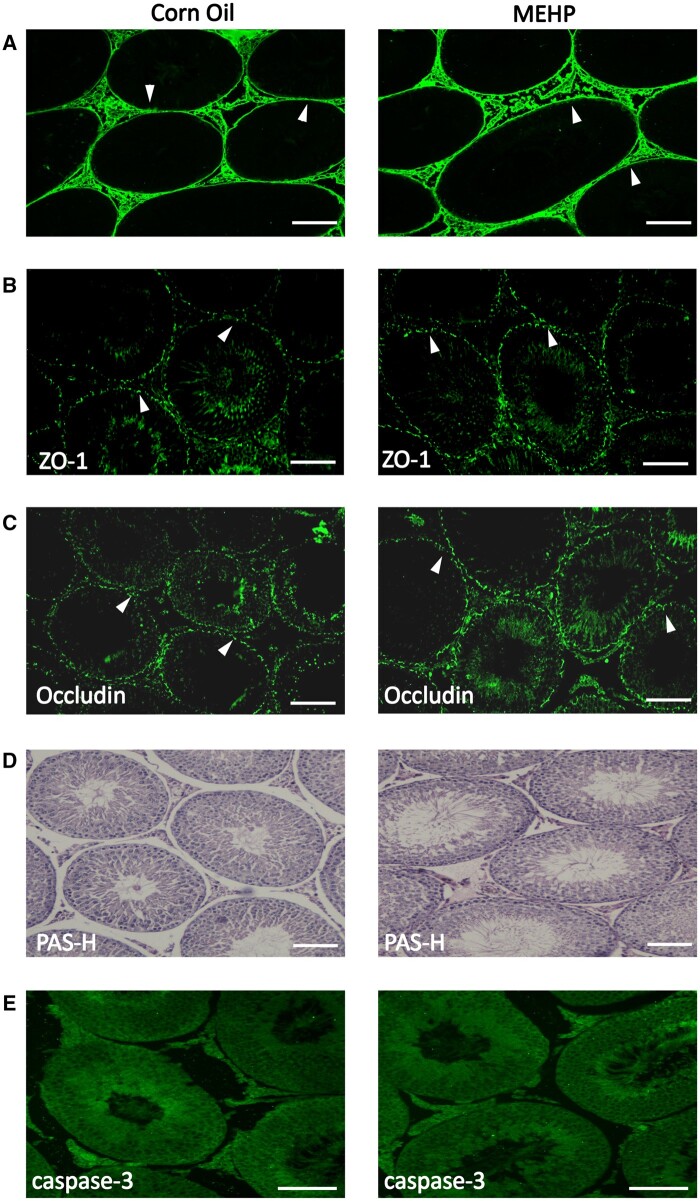
MEHP-induced BTB disruption is reversible. Male PND 28 Fisher rats were treated with either corn oil or MEHP (0.7g/kg) for 24 h, followed by a 5-week recovery period. (A) Biotin tracer assay shows that biotin was restricted to only the basal area of seminiferous tubules (indicated by arrowheads) of corn oil and MEHP-treated prepubertal rats. Immunofluorescent staining of a cross-section of the testis of corn oil and MEHP rats reveal that ZO-1 (B) and occludin (C) formed a similar bright linear pattern in the basal area of seminiferous tubules in both groups (indicated by arrowheads). PAS-H staining (D) of testis sections show normal spermatogenesis in both the corn oil and MEHP group. Immunofluorescent staining of Caspase 3 for both corn oil and MEHP group (E). Positively stained apoptotic cells are indicated by arrowheads. Images are captured with 20× objective and representative of *n* = 7 for each treatment. Scale bars = 50 μm.

To determine if BTB disruption by MEHP treatment could be reversed, PND 27 male Fischer F344 rats were treated with a single oral dose of MEHP or an equivalent volume of vehicle followed by a 5-week recovery period. No biotin infiltrated into the lumen of the seminiferous tubules in either the control group or the MEHP treated followed by 5 weeks of recovery pre pubertal rats, as seen in Figure 5A. This data establishes that BTB disruption induced by MEHP is reversible.

Immunofluorescent staining data of tight junctions (Occludin and ZO-1) in Figures 5B and 5C confirms the integrity of BTB in both the control group and MEHP treated followed by 5 weeks recovery group. Both occludin and ZO-1 were localized in a linear and continuous pattern in the basal compartment of seminiferous tubules in both groups, further validating that MEHP induces reversible BTB disruption.

Histopathology data (PAS-H staining) reveals that the damaging effects of MEHP are reversible as spermatogenesis appears to be normal in pre-pubertal rats 5 weeks after exposure (Fig. 5D). Caspase 3 staining shows no increased apoptosis of germ cells in MEHP-treated group (Fig. 5E).

## Discussion

The potential impact of phthalates on the BTB has raised concerns within the field of reproductive health. BTB is a crucial physiological structure that separates the circulating blood from the testicular microenvironment, safeguarding the developing sperm cells from harmful substances. Studies suggest that exposure to specific toxicants may disrupt this barrier, compromising its integrity (Cavicchia *et al.*, 1996; Cheng *et al.*, 2011). This disruption can increase permeability, allowing harmful molecules to infiltrate the testicular environment and potentially induce inflammation or oxidative stress. These effects may disrupt normal spermatogenesis, leading to male reproductive issues, such as reduced sperm quality and fertility (Sobarzo *et al.*, 2015). While more research is needed to fully understand the extent of phthalate-induced disruption of the BTB, these findings underscore the importance of minimizing phthalate exposure in our daily lives to safeguard male reproductive health.

The SCs of the testis seminiferous epithelium are well understood to play a key role in the creation of tight junctions between adjacent SCs, resulting ultimately in the formation of the BTB and, as a result, creating the specialized adluminal microenvironment required for the development of spermatocytes and spermatids. The completion of the BTB occurs early in animals during the postnatal period before the development of immunotolerance (Bergmann and Dierichs, 1983). Therefore, the BTB also protects the germ cells from being recognized as foreign by the cells of the immune system that develop during the later postnatal period (Mruk and Cheng, 2015).

MEHP has been shown to disrupt the function of SCs in experimental animal models. This disruption results in the loss of spermatocytes by apoptosis, with peripubertal-aged animals more susceptible to the effects of phthalates than adult-aged animals (Murphy *et al.*, 2014; Voss *et al.*, 2018). Previous studies have reported that chronic exposure to DEHP in young rats and mice leads to reduced expression of gap and tight junctions (Yi *et al.*, 2018; Zhao *et al.*, 2023). Our lab has previously reported a loss of spermatocytes and the influx of macrophages following acute MEHP exposure in pubertal but not adult rats (Murphy *et al.*, 2014). One of the possible hypotheses for this observation is that MEHP disrupts BTB in pubertal rats, leading to leakage of germ cell antigens, which in turn causes infiltration of macrophages for repair and recovery of spermatogenesis. However, no studies have explored the effect of acute MEHP exposure on BTB in pubertal and adult rats. Here, we describe the influence of acute exposure to MEHP in male Fischer rats on the functional integrity of the BTB and its reversibility, as well as provide insights into the molecular and cellular signaling pathways that may be involved in the pathogenic mechanism for MEHP-induced disruption of the functional integrity of the BTB.

This is the first report to show a single acute dose of MEHP was shown to compromise the integrity of BTB 24 h after exposure, as seen by biotin tracer infiltrating into the seminiferous epithelium only in pubertal but not adult rats. We further investigated if this disruption of BTB was transient. Pubertal rats were exposed to an acute dose of MEHP and, followed by a 5 weeks of recovery period, showed complete recovery of BTB; hence, this disruption was reversible.

Furthermore, the immunofluorescence pattern of tight junctions (ZO-1 and occludin) was shown to be discontinuous and delocalized compared to the corn oil-treated group. Our findings are consistent with a previous report (Yao *et al.*, 2010; Zhang *et al.*, 2008) that depicted MEHP exposure in 2-compartment cultures has been shown to reduce expression levels of ZO-1 and occludin. Accordingly, Sobarzo *et al.* (2009) have reported that DEHP exposure in 27-day-old rats leads to similar reduced expression levels of tight junctions. These results conclude that disruptions in the tight junction expression levels lead to compromised integrity of BTB. To the best of our knowledge, this is the first study to indicate that acute exposure to MEHP leads to functional disruption of BTB, as shown by the biotin tracer assay.

To further explore the underlying mechanism of BTB disruption, we explored the ERK/MAPK signaling pathway and found that MEHP treatment of pre-pubertal rats induced significant transient activation of MAPK/ERK as well as JNK and p38 phosphorylation levels. The previously published report demonstrated that MEHP exposure in rat SCs leads to activation of p44/p42 MAPK but not SAPK/JNK and p38 (Chiba *et al.*, 2012). Furthermore, treatment with MAPK inhibitor leads to inhibition of MEHP-induced downregulation of occludin and claudin 11, confirming that the MAPK signaling pathway regulates tight junction. Here we report the novel finding that acute MEHP exposure leads to activation of phosphorylation levels of SAPK/JNK and p38. Interestingly, this activation is transient as it initiates as early as 3 hours, peaks at 6 h, and descends back to normal levels by 12 h. Similarly, it was reported that BPA downregulated occludin and ZO-1 via p44/42 MAPK activation pathway (Li *et al.*, 2009). These reports indicate that upregulation of p44/42 MAPK signal transduction may negatively impact spermatogenesis. Other molecular mechanisms have been implicated in phthalates regulation of BTB. Yi *et al.* (2018) have reported DEHP chronic exposure in pubertal rats destroys BTB via excessive ROS-mediated autophagy. Additional reports suggest that the oxidative stress signaling mechanism is involved in adherent cell junction disruption induced by MEHP in SC cultures (Sobarzo *et al.*, 2015).

For the first time, this study identified that acute exposure to MEHP during the pubertal period in rats causes functional disruption of BTB integrity, possibly via a transient surge in p44/42 MAPK JNK p38 signal transduction. We are the first to report that this BTB disruption can be reversed after acute MEHP exposure. We have previously shown that acute MEHP exposure in pubertal rats leads to a transient influx of macrophages and neutrophils (peaks at 12 h) (Murphy *et al.*, 2014). Recently, we reported that acute MEHP exposure in pubertal rats leads to massive infiltration of peritubular macrophages, which are hypothesized to significantly stimulate the spermatogonium within the stem cell niche (Gillette *et al.*, 2021). Since BTB plays a crucial role in developing immunotolerance, a disruption of the BTB may allow for the leakage of GC antigens and signal peritubular macrophages to replenish the lost germ cells by stimulating spermatogenesis. Investigations to decipher the functional role of the peritubular macrophages in the repair and resolution of spermatogenesis in the testis are ongoing.

## Declaration of conflicting interests

The authors declared no potential conflicts of interest with respect to the research, authorship, and/or publication of this article.
